# Pregnant women’s navigation of information on everyday household chemicals: phthalates as a case study

**DOI:** 10.1186/s12884-015-0748-0

**Published:** 2015-11-25

**Authors:** Justin M. Ashley, Alexandra Hodgson, Sapna Sharma, Jeff Nisker

**Affiliations:** Department of Obstetrics and Gynaecology, Schulich School of Medicine & Dentistry, Western University, London, ON Canada; Children’s Health Research Institute, Western University, London, ON Canada; London Health Sciences Centre, Victoria Hospital, Rm E2-620E, 800 Commissioners Rd., E., London, ON Canada, N6A 5W9

**Keywords:** Pregnant women, Household chemicals, Phthalates, Navigating risk

## Abstract

**Background:**

Current developments in science and the media have now placed pregnant women in a precarious situation as they are charged with the responsibility to navigate through information sources to make the best decisions for her pregnancy. Yet little is known regarding how pregnant women want to receive and use health information in general, let alone information regarding the uncertain risks to pregnancy in everyday household products such as phthalates found in cosmetics and canned food liners. Using phthalates as an example, this study investigated how pregnant women obtain, evaluate, and act on information regarding their pregnancy.

**Methods:**

Pregnant women were recruited using pamphlets and posters distributed in prenatal clinics, prenatal fairs and physician offices in Southwestern Ontario Canada. Research participants were engaged in 20 to 40 min semi-structured interviews regarding their use of information sources in pregnancy, particularly regarding phthalates in cosmetics and canned food liners. Interviews were transcribed verbatim and analyzed using constructivist grounded theory techniques supported by NVivo 9™ software.

**Results:**

Theoretical sufficiency was reached after 23 pregnant women were interviewed and their transcripts analyzed. Three overlapping themes resulted from the co-constructed analysis: I-Strength of Information Sources; II-Value Modifiers; and III-Deciding to Control Exposure. The research participants reported receiving information from a wide range of sources that they perceived varying in strength or believability. They then described the strategies employed to increase the validity of the message in order to avoid risk exposure. Pregnant women preferred a strong source of information such as physician, government but frequently used weak sources such as the internet or the opinions of friends. A model was developed from the relationship between themes that describes how pregnant women navigate the multiple sources of information available to them.

**Conclusion:**

Our study provides insight into how pregnant women receive, appraise, and act on information regarding everyday household chemicals. Clinicians and their professional organizations should produce specific educational materials to assist women in understanding exposure to everyday products in pregnancy.

## Background

It is challenging for pregnant women to understand the risks of and avoid exposure to everyday household chemicals [1, 2], because of the ubiquitous and complex nature of these chemicals [3]. The uncertainty of the effects of many household chemicals on human pregnancy makes it difficult for health professionals to provide meaningful counseling regarding these exposures [4]. Phthalates are a group of chemicals often found in cosmetics [5, 6] and liners of canned food [5]. They have been associated in the human with premature birth [7, 8] and increased chance of allergies and asthma [9–11]. Phthalates have also been found to effect the human male reproductive system [9], including reduced penile size [9, 12], cryptorchidism [12], and shorter anogenital distance [13].

Phthalates are listed among a group of environmental toxins that are thought to cause endocrine disruption [2, 9, 14–17]. In 2013, the Royal College of Obstetricians and Gynaecologists in their Scientific Impact Paper on “Chemical Exposures During Pregnancy” [2] raised concerned about phthalates. Similar sentiments were expressed by the American College of Obstetricians and Gynecologists with the American Society for Reproductive Medicine in their joint Committee Opinion on “Exposure to Toxic Environmental Agents” [17]. Yet because the uncertainty of the effects of phthalates continues, clinicians do not include discussion of phthalates in prenatal and preconception care, nor have their professional organizations directed them to do so. Recent work suggests that health professionals do not possess the time, knowledge or training to provide meaningful counseling on chemicals that cause endocrine disruption in general and phthalates in particular [18].

Women are being increasingly warned in the lay press of the harmful effects of phthalates [19–23], including Glamour magazine [21] that recognized phthalates as one of three chemicals women need to avoid. Current developments in science, medicine, and the media have now placed pregnant women and women contemplating pregnancy in a precarious situation as they are charged with the responsibility to navigate through information sources to make the best decisions for their pregnancy [24–26]. Yet little is known regarding how pregnant women and women contemplating pregnancy want to receive and use health information in general [27–31], let alone information regarding the uncertain risks to pregnancy in products that they use every day.

Using phthalates as an example of an everyday household chemical, this study explored how pregnant women obtain, evaluate, and act on information regarding their pregnancy.

## Methods

### Recruitment

Purposeful sampling was used to recruit participants who would best address the research question. Posters and pamphlets were distributed in clinics, prenatal fairs, and community centers in Southwestern Ontario Canada. After being presented with the pamphlets, participants had the opportunity to approach the researchers if they wished to participate or had more questions about the study in general.

In order to elicit a wide range of perspectives and understandings, very few inclusion and exclusion criteria were applied. Women had to be pregnant at the time of recruitment, English speaking, and 18 years of age or older. These criteria were put in place to help facilitate the informed consent process as well as ensure the participants could appreciate some of the complexity regarding phthalates. Of note, patient characteristics and demographics were not charted as the researchers felt this was a barrier to developing rapport and creating an honest and open environment during interviews.

Recruitment continued until theoretical sufficiency was reached [32]. That is to say that analysis was no longer revealing new patterns, concepts, and insights from the empirical data [33]. Institutional Review Board ethics approval was obtained through the Western University Health Science Research Ethics Board (17406E).

### Interviews

Interviews were conducted in London, Chatham, Sarnia, and Walkerton. To help ensure voluntary participation, participants were given a verbal explanation of the project, a written description of the research, and an opportunity to ask any questions they might have had prior to signing an informed consent form. The participants engaged in 20 to 40 min semi-structured interviews with two members of the research team. During these interviews, ten non-leading prompts (Table 1) were used to stimulate conversation and elicit participant’s unique perceptions, understandings, and experiences. The prompts were refined throughout the research process as new insights pointed to more effective ways to elicit rich and meaningful data.

**Table 1:** Sample prompts to be used in interviews with pregnant women

1.What does risk in pregnancy mean to you?
2.Do you think environmental exposures could impact your health?
3.Tell me about your access to resources throughout the pregnancy
4.Have you heard about Phthalates?
5.***Provide Patient with Information***
6.What else, if anything, would you like to know about phthalates?
7.What steps, if any, could you take to control your exposure to phthalates?
8.What role/responsibilities, if any, do your obstetrical care providers have regarding your health and health of your fetus?
9.Is there a point at which you think various parties should be providing you or the general public with information regarding potential risk? Why? Please expand.
10.If you knew about Phthalates what you know about other risks in pregnancy, would it change your behavior?

The interview consisted of three interrelated and iterative sections. The beginning of the interview focused on participant’s experiences with risks in pregnancy, their perceptions of household chemical risks, and their access to resources regarding risk. The second section of the interview involved providing general information regarding phthalates. Information regarding sources of exposure and potential health outcomes were provided on a brochure. The brochure read:

Phthalates are compounds that are used to make plastics flexible in their final applications. They are used in floor tiles, clothes, medical supplies, toys, food packaging, and personal care products. These compounds have also been shown to leach out of various products, and are also present in appreciable amounts in our environment…..mimic naturally occurring hormones in the body, interfering with the endocrine system to produce adverse developmental and reproductive effects. However, the full range and extent of these effects have not yet been identified.

The final section of the interview focused on participants’ understandings of the risk of phthalates, how exposures may affect pregnancies, and what other information would be helpful for them to know. Finally, the participants were offered an opportunity to ask their own questions, clarify misunderstandings, or share any remaining thoughts. The pacing of the interview was determined by the richness of the participants’ responses and the overall flow and atmosphere of the interview.

The interviews were audiotaped and transcribed verbatim including pauses and notes on the emotional tone of the spoken text by a professional transcriptionist. All identifying information was removed from the transcripts. Extensive field notes were taken before, during, and after interviews to capture context which would inform the analysis process.

### Analysis

Constructionist grounded theory as outlined by Charmaz [32] was used to analyze the data and develop a middle range theory. Analysis was supported with NVivo 9^TM^ software (QSR International Pty Ltd, Doncaster, Vic, Australia). Each transcript was read in its entirety to determine what was said before engaging in initial coding. Then transcripts were independently coded by at least two researchers. Analysis began with a phase of initial coding where the data was broken into small, salient codes, using a segment-by-segment coding strategy. The complexities of research participants’ decision-making processes, apparent during the initial coding phase, were broken down into simpler concepts. By fragmenting the data, individual ideas offered by participants were considered and re-organized in a framework that simplified decision making processes and provided the foundation for the focused coding stage. Transcripts were analyzed independently by JA, AH and SS and then discussed collectively among all researchers. These discussions were used to identify common themes researchers identified, discuss potential theoretical directions, and develop a more refined coding scheme before transitioning to the focused coding phase. The focused coding phase involved revisiting the transcripts and re-organizing the data with the higher level coding scheme while still being open to new potential theoretical and thematic directions [32].

Analytical memos were used throughout the process to capture the researchers’ ideas as well as chart various theoretical directions. During initial coding, memos were often written to describe emerging codes and consider relationships between them. As the analysis reached the focused coding phase, the memos were used to explore the theoretical relationships between these codes. This involved diagramming relationships between codes with brief notes regarding the function of these relationships. Memos were also used as an opportunity to be reflexive about the analysis and understand how the researchers were contributing to the co-construction of the data [32]. The memos often dealt with representing the participants’ thoughts and perceptions honestly and understanding how the researchers’ experiences may have influenced the research.

The research process was ongoing and iterative as researchers constantly reviewed and re-reviewed data, considered new theoretical directions, and conducted more interviews as needed. Through this process, a model depicting how women receive, appraise and act on risk information was co-constructed from the data.

## Results

**Fig. 1:**
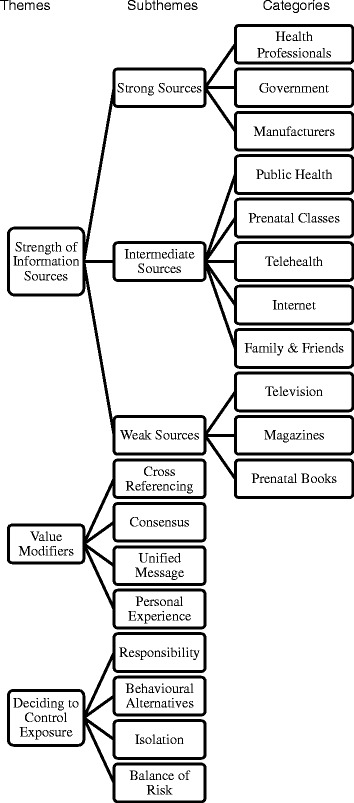
Derivation of themes from subthemes and categories

Twenty-three pregnant women were recruited before theoretical sufficiency was reached as the researchers felt that new data was no longer revealing new insight into how pregnant women engage with new information. Three broad and overlapping themes were co-constructed from the analysis outlined in Fig. 1.

### Theme I - Strength of information sources

Research participants discussed receiving information regarding risk in pregnancy from a wide range of sources including family, television, internet, pamphlets, physicians, and telehealth. However, each information source was not considered equal. Instead, women perceived information existing on a continuum of inherent strength from weak to strong. In the context of the research, “strength” was a loaded term which often varied between research participants. It should be noted that this continuum is fluid, individualized, and often context dependent. Most frequently, the strength of a source was seen as a function of a participant’s perceptions of the trustworthiness of the source, the expectation that the information provided was accurate, and the amount of work needed to verify or modify the information (see Theme II – Value Modification). The strength of a specific source was determined primarily by what was said in the transcripts. However, additional input from field notes, memos, and team discussion influenced the determination of what ultimately was designated as a strong, intermediate, or weak source. Research participants’ comments speak to perceived strength of sources from which information is received. Theme I is comprised of three subthemes: IA-Strong sources; IB-Intermediate sources; and IC-Weak sources.

### IA-Strong sources

Examples of strong sources often included physicians, other health professionals, professional bodies and governments. For the research participants, being counseled about a particular risk from a health professional or receiving a public health message from the government meant that a particular risk was significant and should likely be avoided as suggested by comments of research participants RP 3, RP5, and RP11.

*I would believe that the healthcare provider could help give an insight to it if, ah, no one else, because the healthcare provider’s role is to maintain your standard, right, and keep you healthy and unfortunately that’s the only person that’s going to be able to do it. – RP3*

*I’d rather them, yeah, tell me everything, honestly too, you know, and then I also think too though that if they know it will harm then it’s, they’re responsible to inform us and to direct us to take that harms out of way, cause what’s the sense of growing someone not to their full potential, I don’t get that, why would you shorthand your people, like*. – *RP5*

*I think their* [health professional] *role is very important and they need to tell you everything even if you, don’t want to hear it or you’re nervous to hear about it, I think you should be told every kind of risk because they don’t know exactly your living environment is, or what you do, where you go, people you’re around with. – RP11*

RP18 considered public health boards to be knowledgeable about risks in society are and inform the public:

*The public health I think should be there by the, provide the information on everybody’s general health, whereas my doctor is specifically for me. And this would be something for, like, everybody’s general health*. –*RP18*

Interestingly, some research participants perceived manufacturers as strong sources in that they believed the government would not have allowed the product and information on the package unless it was safe. This was suggested in the comments of RP21.

*Like, I would, if there’s a product on the shelf in Canada I would think it’s safe to use. Like I shouldn’t have to take it* [laughs] *everything I buy and say, is this safe or what. I don’t and maybe I should question more but I don’t. I just take it for granted that… –RP21*

Ultimately, messages from strong sources were seen as legitimate by research participants. However, the perceived strength of an information source was only the initial factor as to whether a woman chose to act on the information from the strong source.

### IB-Intermediate sources

Research participants commented on a second group of information sources that they often considered important but felt they lacked the trustworthiness and accuracy of strong sources. These intermediate strength information sources included prenatal classes, telehealth, and the Internet. Although frequently accessed, the research participants exhibited caution when using these sources as evidenced by the comments of RP5 and RP17.

*So they’ll tell you whatever to scare you away from one person’s stuff and say, oh, ours is wonderful, you know, but I don’t, you don’t really find places that just tell it as it is, you know, except for Wikipedia and Google, but again, like I said with Google you know, you might click the third one down and the two above might have more information, you never know, so it’s still kinda bits and pieces.* – *RP5*

*I usually call Tele-health if I want to know stuff. Um, oh, they can be, very really helpful or overly useless. So it’s one extreme to the other. But it’s worth a shot. If you go to the doctor’s the next day and they say a completely different thing then you just wasted your time trying to do that*… - *RP17*

### IC-Weak sources

Weak sources included media sources such as magazines, television, and self-help books on pregnancy. Research participants discussed being concerned by the potential inaccuracies and exaggeration of truth in these information sources. However, weak sources were considered still valuable by research participants as it was often their first exposure to learning about risks as evidenced by RP15and RP20.

*… I don’t use anything of the media to portray the fact, but it’s a good way to spark my interest on something. So if I read it in an article or something, oh that’s interesting, but I’m not going to take what they had as the gospel. –RP15*

*My husband brought up, maybe something in the diapers, I think we heard somewhere that a product in the diapers could maybe be increasing prostate or ovarian cancer or something like that…And because I haven’t heard anything aside from someone’s opinion, like, I think it came up in a conversation, I haven’t heard anything to back it up. So I haven’t thought about it further…* - *RP20*

### Theme II - Value modifiers

Research participants discussed employing various techniques to fortify the strength of information they were receiving from information sources before fully considering whether to engage in risk avoidance behaviors. This was particularly true of sources that arose from the Internet. Their comments in this regard led to Theme II - Value Modifiers (Fig. 1). Theme II is comprised of four subthemes: IIA–Cross Referencing, IIB–Consensus; IIC–Unified Message; and IID–Personal Experience. Research participants described these four subthemes as interrelated processes used to increase or decrease the value of any particular information source.

### IIA–Cross referencing

Cross referencing was a simple but commonly used method that research participants used to augment the quality of a weak source. Women would often double check the information supplied by a weak source (e.g., a news report) with a strong source (e.g., physician or government) before taking it seriously, as illustrated by the comment of RP13.

*Um, I’m usually just, like, happy with what the doctor says but if it was something that I’d read on the Internet then I would, that’s something I would ask the doctor about it, but if the doctor had said it in the first place I wouldn’t go check on the Internet. –RP13*

Similarly, a strong source could endorse a weak source such as a pamphlet or website which would then give that weak source the strength of a strong source. As evidenced by the comments of RP20.

*Maybe even to be given a resource that they trust would be good, like a, like if they have…a book that they could recommend, that would be helpful because they don’t have a lot of time to go over everything. –RP20*

### IIB–Consensus

Research participants acknowledged these sources as useful and easily accessible, but also were very aware of the varying quality of these sources. This often pertained to internet searching as women tried to make sense of information on their own. Participants often discussed reconciling multiple and often conflicting information sources either by developing a consensus amongst the sources or taking a moderate stance between opposing views. These techniques were often used for intermediate strength information sources such as the internet, telehealth, and prenatal classes.

*I go to more than one* [website], *like I don’t just take one person’s word for it… If it’s one of those websites where it’s a lot of people explaining it then it’s usually more believable than, you know. –RP13*

*I usually look at a bunch of different ones* [websites] *and then from those like basically, like, you can tell which ones are one way and the other way and I kind of like to be in the middle –RP14*

### IIC-Unified message

Pregnant women often looked to strong sources to provide a unified message about risk information. Conflicting health information from strong sources had the effect of potentially depreciating the value of the health message and affected pregnant women’s confidence in decision making.

*Cause there are so many mixed messages about everything in pregnancy. So, like one doctor one thing, another doctor says another thing, you obviously go with what your doctor says, but something like this where there’s a unified message should come from a public health board or something like that, or even doctors. –RP19*

### IID-Personal experience

Probably the most significant value modifier identified by research participants was personal experience. Research participants drew upon their lived realities, previous pregnancies, and their jobs to make the final determination about the strength of a piece of information. For example, RP20 was on her third pregnancy and had become increasingly less worried about various exposures in pregnancy as her previous two pregnancies were healthy.

RP20 described being more relaxed about risk relative to her first pregnancy:

*Um, again with my first pregnancy I was probably, like a 10 out of 10 worried about everything, like what can I eat, what can’t I eat, um, you know, even if I was, like just the gas cap off the lawnmower one day, and I was filling it up, and I got a little bit of gas on my hands, I went in to wash it off, and I was worried, Oh no!, I touched the gas, you know, and, um, just cause you’re, you’re not sure what can hurt the baby. And people were that’s a little excessive…I have definitely calmed down in terms of how worried I am now*. – RP20

However, another participant, RP23, had become increasingly more worried as she lost her second pregnancy in a car accident.

*My first one* [pregnancy] *I was, I was fine, like, I heard some horror stories but there’s always horror stories, and then I got in a car accident and lost my second pregnancy. And then my third one was the gastroschisis and now this one I’m like something’s going to go wrong, you know, I need to … I don’t know, it’s scary… –RP23*

### Theme III-Deciding to control exposure

Although an information source regarding a particular risk was perceived as significant, research participants did not immediately adopt the corresponding risk-avoidance behaviour. Instead, research participants discussed considering the legitimacy of the risk in the context of their life, the practicality of adopting that change, and the potential emotional repercussions. Theme III - Deciding to Control Exposure outlines the interrelated processes participants described when deciding what risks are worth avoiding and which risk avoidance strategy to engage in. Theme III is comprised of four subthemes: IIIA-Responsibility; IIIB- Behavioural Alternatives; IIIC-Isolation;; and IIID-Balance of risk.

### IIIA-Responsibility

Research participants discussed the importance of taking on the responsibility of evaluating the relevance of a particular risk and the importance of adopting a change as seen in the comments by RP4 and RP8.

*I believe you should be researching it yourself because your obstetrician doesn’t have time to know, like, everyday products that you are using, which brand of product, and to be researching everything herself…she should have a knowledge, but she doesn’t have knowledge on every single product. –RP4*

*Every time something comes up that I don’t understand I just try and, try and figure out, ah, what, what it means, how to prevent it, or how to make it better, different things like that, I’ve had a lot of little problems along the way through the pregnancy, so… –RP8*

Being constantly vigilant and responsible often took an emotional toll. Research participants discussed feeling overwhelmed with the amount of information to sift through and the potential implications of their decisions. These feelings are illustrated in RP22’s comments.

*Ah, I find it can be very overwhelming, especially being our first child, not only the fact that it’s in me but also that, you know, you’re responsibility for a living being 24/7 in a few months, so yes, it can be very overwhelming. –RP22*

### IIIB-Behavioural alternatives

In regards to risk avoidance behaviour, most of the participants discussed searching for alternatives to potential house chemical risks. One example, as evidenced by RP5 was to switch from plastic materials to glass.

*I’d rather use glass stuff for, you know, I know it breaks and stuff, but it’s, you know, it’s a little safer, so, why not.–* RP5

Another risk reducing behaviour participants discussed related to alternatives was advanced planning. Participants discussed anticipating and avoiding certain household chemical risks before the pregnancy began. As RP15 explains:

*We haven’t heard a lot about it* [phthalates] *but, what I have heard…I would at least try and position myself, if I’m looking at it as a possible issue, there’s probably some evidence that’s been brought up to have that research initiated, so, I’d probably try to avoid it from day 1*. – RP15

### IIIC-Isolation

The ubiquity of everyday household chemicals was often perceived by women as difficult to meaningfully control their exposure. For some women, this came with a sense of relief, as they felt there was nothing that they could really do and should not worry about it. As RP20 explains:

*I guess so, yeah, and realizing we can’t control everything, there’s some things that you have to do, and, that the baby’s going to turn out okay.* [laughs] *…I think it’s a little liberating knowing that, er, a little freeing, you know, using your mind to realize you can’t control everything*. – RP20

Other women, such as RP12, could not help but feel isolated as a result of these behaviours:

*I don’t know, it isolates yourself and when you’re trying not to be around people, or do things, you end up isolating yourself a little more. Um, definitely I feel better all going out, even if it’s not to a club but you’re all at somebody’s house, you know, our friends smoke in their home… don’t go to their house right now, cause their house is a big smoky mess, so it’s not, I won’t go there. –RP12*

RP23 also experienced a frustration with people who smoked around her in public:

*I don’t know, I don’t understand, people … they don’t understand that you don’t wanna sit next to someone who’s smoking a cigar or something, like, like if you’re out or whatever, it’s just, I don’t know. You take more precautions… I wouldn’t* [normally] *care if we had to wait for someone to finish their smoke or whatever, but if I was pregnant I don’t appreciate people smoking around me*. – RP23

### IIID-Balance of risk

Finally, one of the most important determinants of whether or not participants would adopt a new risk avoidance behaviour was balanced against the perceived threat of the risk. Most women had a limit as to how far they were willing to make changes in their lifestyle to avoid certain risks. Although the risk of phthalates was perceived by participants as legitimate, the ubiquitous nature of household chemicals was often perceived as risks participants were willing to accept. Women, such as RP16, often struggled to find a balance between cost, convenience and the potentially detrimental effects of phthalates. As she explains:

*Holy, I don’t know.* [laughter] *I don’t know, because, I mean … you need it* [phthalates] *on one hand…but on the other hand, you know, well I guess it should be known that there is a concern in regards to the chemical that is being used and leave it up to the individual whether or not, you know, let them, I guess, be known it’s a risk… –RP16*

Similarly, RP19 discussed finding a balance between being exposed to risks of phthalates and the financial burden of purchasing phthalate-free products.

*You’re talking $5 versus $100 then, and if there was an unknown risk, then I would have to weigh that out, but if there was a known risk then, then how can you … you have to do what you have to do. –RP19*

Cumulatively, the strategies pregnant women used to determine the relevance of information were diverse, interrelated, and apparently not without emotional repercussion. Subsequently so are the ways on which they act on risk information.

### Model - How pregnant women navigate information on everyday household chemicals

**Fig. 2:**
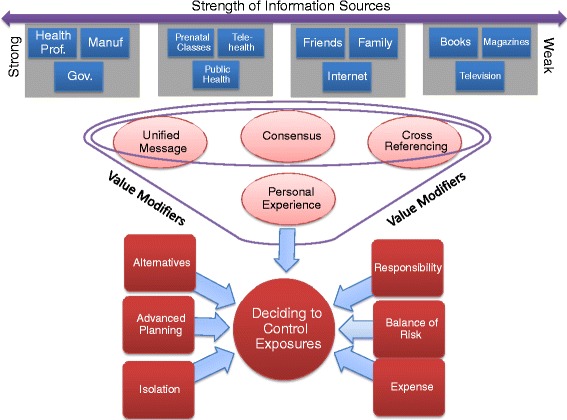
Model of how pregnant women navigate information on everyday household products such as phthalates

A model (Fig. 2) was developed from our research to conceptualize how pregnant women engage with, internalize and act on information concerning everyday household chemicals. The variety of information sources research participants discussed and their perceived strength (Theme I) was laid out on a continuum at the top of the model with increasing strength from left to right. Once an information source was canvassed, research participants began to modify or augment the inherent strength of that source through the use of the four techniques outlined in Theme II. Each value modifying technique (Subthemes IIA-D) is placed on the model closest to the types of information sources where participants described most frequently utilizing that technique. Importantly, Subtheme IID – Personal Experience is placed at the bottom as it was often used in all instances as a final appraisal of information. Finally, Theme II-Value Modifiers is depicted on the model as a funnel to represent the appraising of information and the funneling of only the most significant information to the final stage of the model. Once a piece of information is deemed significant, they decide whether they actually can avoid an exposure and how they would do so (Theme III). Factors pregnant women discussed, including responsibility, balancing risks, financial cost, isolation, avoidance, and advanced planning are depicted as inputs in the decision making process. Participants made decisions through an iterative and ongoing process of balancing new information with practicalities of adopting that change.

## Discussion

The increasing media and political attention regarding household chemicals such as phthalates pose challenges to pregnant women and their obstetrical care providers. This study helped develop an appreciation of how pregnant women receive, appraise, and act on new information regarding everyday household chemicals such as phthalates. Women identified healthcare professionals and the government as “strong sources” of information that when available their message regarding a specific risk, could only be modified slightly by weaker sources such as media, family and friends. To a lesser extent, manufacturers were also perceived as strong, as it was believed they would not be allowed to sell their products unless they were deemed safe by the government. Women described how they would check a variety of information sources, their perception of the strength of the source, and then how they would modify the value of the information by engaging in various techniques including cross referencing and using personal experiences. In determining how they would act on the overall information on avoidance of potential risks of everyday household chemicals, pregnant women considered factors such as financial cost, practicality of adopting change, and responsibility.

Pregnant women felt more confident receiving information from strong sources such as physicians regarding everyday household chemicals. However, barriers to accessing strong sources may cause pregnant women to access weaker sources such as the media or friends even though they are aware of their inherent weakness. Barriers to receiving information from strong sources such as physicians include current lack of concern among physicians about everyday household products such as phthalates [18], as well as their time limitation in providing preconception and prenatal counseling [18, 29, 30]. The lack of proven risk may discourage both the unsolicited provision of information from physicians and pregnant women’s ability to ask questions about phthalates and other household chemicals, subsequently inhibiting the pregnant woman’s comfort in informed decision-making.

Uncertainty of risk also makes it difficult for health professionals, professional organizations, and governments to make meaningful policies [34] that clinicians can use to frame discussion with pregnant women. In 2013, the Royal College of Obstetricians and Gynaecologists produced a Scientific Impact Paper on “Chemical Exposures During Pregnancy: Dealing with Potential, but Unproven, Risks to Child Health” [2] which included a list of products women might choose to avoid if they are pregnant or contemplating pregnancy in order to reduce potential yet unproven risks [2]. Also in 2013 the American College of Obstetricians and Gynecologists with the American Society for Reproductive Medicine produced a Committee Opinion on exposure to toxic environmental agents in pregnancy [17]. These documents may eventually shift the standard of care to encourage clinicians to discuss phthalates and other everyday household exposures with women during pregnancy and preconception. These documents when accessed on the internet by women who want to learn more about potential household toxins and pregnancy will serve as a “strong source” and likely prompt questions to their clinician who they consider the most important “strong source” of information. However, neither document goes far enough in assisting clinicians in discussing the particular risks of endocrine disruptors such as phthalates and brominated flame retardants and the particular avoidance strategies required to mitigate the particular risks. By providing pregnant women with a pamphlet or a link to a website that is consistently endorsed by health professionals, their national bodies and other “strong sources”, clinicians can provide in depth information on phthalates and other household chemicals without infringing on time they would prefer to spend in discussion of other risks in pregnancy.

The pregnant women interviewed in this study also demonstrated caution with information sources. The subthemes of Cross Referencing, Consensus Building, and Unified Message indicated the increased medicalization of pregnancy and subsequent need for prudence and risks management [35]. However, the research participants’ comments organized in Subtheme IID-Personal Experience indicated that for these women experiences such as previous pregnancies, job training, and family life continue to be strong determinants of the value of information they were receiving and how well it fit with their personal perceptions of their pregnancy [21]. When determining if an everyday risk was important enough to try to control exposure (Theme III) research participants spoke both of their increased scrutiny of health information and the emotional toll of having to decide or not to decide on certain risk avoidance behaviours. Whether or not a woman acted on the information she accrued was often determined by balancing factors such as cost, the ease of avoidance strategies, and the availability of reasonable alternatives. Again, this is consistent with current social pressures for mothers to take on the responsibility to negotiate a wide range of information sources and make decisions in the best interest of her fetus [34, 36].

The women in our study discussed the emotional toll of these avoidance strategies, but felt they were necessary or they could be women at risk of being labeled as a bad mother [25, 36]. Some women felt isolated from their family and friends while other women felt overwhelmed by the amount of information available. Conversely, other women expressed relief by the uncertainties and difficulty of avoiding household chemicals such as phthalates as their risk of avoiding exposure would be less burdened by blame. Regardless, women expressed desire to have knowledge about phthalates and other household chemicals in order to make the appropriate decisions for themselves.

Based on our research into how women understand, obtain, assess and respond to new information about exposures to everyday household chemicals during pregnancy, it appears that a strong-to-weak knowledge dissemination strategy that is consistently encouraged by “strong sources” such as physicians, their professional organizations, and government agencies is preferred. This type of strategy was vividly offered by RP15 during her interview when she proposed the use of a website that was created by the government and endorsed by various health professionals to chart various household chemicals by the certainty of the risk and the severity of the health outcomes. By selecting a particular household chemical the user would be led to a page which offered more detailed information, risk-avoidance strategies that women could engage in, alternative products to buy to decrease exposure, links to original research, and a message forum for women to discuss these risks with health professionals, researchers, and government officials.

### Limitations

The findings in this study were co-constructed [32] between the 23 pregnant women and the research team. The results aim to represent all research participants’ comments and perceptions but may not have been able to do so. This research may not be generalizable beyond the pregnant women interviewed. Given certain logistical and time constraints particular to the research team, member checking was not an appropriate strategy for this project. Instead, other strategies were used to ensure the results reflected the intentions of the research participants. Specifically, findings were compared against field notes originally taken during the interviews. Moreover, researchers had the opportunity to share preliminary results with other mothers as well as the obstetrical community.

Further research is needed to explore women’s experiences of seeking information about everyday household chemical risks in other contexts and geographic locations in order to further develop and potentially validate the proposed model. Subsequently, there is a need to develop and test knowledge dissemination materials based on Fig. 2 to understand how such knowledge of everyday household chemical risks is used by pregnant women and what obstacles they face. Researchers should consider methodologies that would allow for the dissemination of material to continuously be tailored and adapted to the local context.

## Conclusion

This study helped develop a model of how pregnant women receive, appraise, and act on information regarding exposure to household chemicals such as phthalates. The pregnant women participating in this study used a variety of information sources with perceived inherent strength that could be modified by various techniques including cross referencing and personal experiences. Women preferred information from strong sources such as clinicians and governments that could be modified by weak sources such as the internet. When deciding to avoid exposure, pregnant women considered financial cost, practicality, and responsibility. Professional organizations should develop specific public engagement and continuing professional development materials to assist pregnant women and women contemplating pregnancy when making decisions regarding avoidance of phthalates and other everyday household chemicals.
